# Neighborhood Disadvantage and Poor Health: The Consequences of Race, Gender, and Age among Young Adults

**DOI:** 10.3390/ijerph19138107

**Published:** 2022-07-01

**Authors:** C. André Christie-Mizell

**Affiliations:** Department of Sociology, Vanderbilt University, Nashville, TN 37325, USA; andre.christie-mizell@vanderbilt.edu

**Keywords:** race, gender, age, young adulthood, neighborhood disadvantage, self-rated health

## Abstract

The objective of this study is to examine the relationship between neighborhood disadvantage and poor self-rated health for a nationally representative sample of Blacks and Whites in young adulthood, 18 to 30 years old. Data were from 16 waves (1997–2013) of the National Longitudinal Survey of Youth 1997 cohort (*N* = 6820 individuals; observations = 58,901). Utilizing the stress process model and generalized estimating equations to account for the correlated nature of multiple responses over time, results show that neighborhood disadvantage increases the odds of poor health for all groups. This positive association is strongest in the most disadvantaged neighborhoods and is heightened as young adults age. There are also notable race and gender differences. For example, Blacks, who live in the most highly disadvantaged neighborhoods, seem to be somewhat shielded from the most deleterious effects of poor neighborhood conditions compared to their White counterparts. Despite greater proportions of Blacks residing in harsh neighborhood environments, Black men experience better health than all other groups, and the health of Black women is no worse compared to White men or women. Limitations and directions for future research are discussed.

## 1. Introduction

Research on health outcomes has firmly established that residents of disadvantaged neighborhoods (e.g., high crime rates, elevated poverty rates, and few employment opportunities) suffer worse health than their counterparts in more affluent communities [1,2,3]. Disadvantaged neighborhoods are characterized by high levels of poverty, physical dilapidation, a disproportionate number of female-headed households, high unemployment, and alarming rates of crime [2]. The social disorder inherent in disadvantaged neighborhoods inhibits health-promoting resources and behaviors, including economic stability, access to healthy foods, physical exercise, and psychological safety [2,3,4]. In poor communities, scholars observ higher rates of chronic conditions (e.g., obesity, asthma), depression, anxiety, sexually transmitted diseases, cigarette smoking, illicit drug use, and alcohol problems [1,5,6,7].

Recent data from the U.S. Census Bureau show that 24 million U.S. residents live in impoverished, disadvantaged neighborhoods [8]. These are neighborhoods in which one-fifth of all community members live below the federal poverty line [8,9]. Racial and ethnic minorities are more likely than Whites are to live in these areas. For example, 20.9% of Black Americans reside in disadvantaged neighborhoods compared to only 4.3% of their White counterparts [8,9,10]. Therefore, compared to Whites, Blacks are at greater risk for the negative health consequences associated with deprived neighborhood conditions. Moreover, poor neighborhoods threaten the health of women more than men [11,12]. Studies show that, in disadvantaged neighborhoods, women are at greater risk for a host of mental and physical health problems, and are more likely to experience violence and mortality [11,12,13]. Further, a constant feature of impoverished neighborhoods is the disproportionate numbers of mother-headed households, which are the poorest households in the U.S. [11].

The same research literature that pinpoints race and gender differences also implicates age as a factor in how neighborhoods are connected to the risk for poor health [14,15]. For instance, older adults may face more vulnerabilities to mental and physical health because of exposure to harsh neighborhood conditions over time [14,15,16]. On the other end of the age spectrum, there has been considerable attention to child outcomes. These studies generally find that the healthy development of children is challenged in disadvantaged neighborhoods due to less physically safe environments, family economic deprivation, high crime rates, and a lack of access to quality health care [17,18,19,20]. Despite the strength of these findings for the elderly and children, less is known about how the health effects of neighborhood conditions vary at other points in the life course.

In this study, I focus on the impact of neighborhood disadvantage on the self-rated health of young adults aged 18–30. As a subjective measure, self-rated health captures dimensions of both physical and mental health [21,22,23]. Numerous validation studies show that measures of self-rated health are reliable. Moreover, such measures are reliable regardless of race, ethnicity, gender, age, and country [24,25,26,27,28,29]. Young adulthood is a unique life stage in which individuals are taking on new roles (e.g., employment, marriage, and parenthood), individuating from families of origin, and developing an adult sense of self [30,31]. The processes associated with young adulthood are stress-inducing, and I examine whether race–gender status combined with neighborhood disadvantage puts some individuals at greater risk for poor health during this period. Beyond the focus on race–gender status and neighborhood conditions, I also investigate whether and how age matters among young adults. That is, I pay particular attention to whether the relationship between neighborhood disadvantage and health by race–gender status is the same, for example, for 18-year-olds compared to 25- and 30-year-olds.

The current study offers three innovations to the research literature. First, this exploration includes longitudinal data for a nationally representative sample of U.S. Blacks and Whites with enough variation to carefully explore race–gender differences. This work offers a level of generalizability not found in more localized samples. Second, this study focuses on young adulthood, a stage of the life course that has received less attention than older adulthood, adolescence, or childhood. While there is an expectation that young adults are relatively healthy compared to their older counterparts, whether and how poor neighborhood conditions are associated with less favorable health may add to our understanding of how certain groups accumulate health disadvantages in this part of the life course. Third, while young adulthood is often conceptualized as a single stage in the life course, this study takes seriously the need to better understand variations in how different groups age into young adulthood [32,33]. Expectation and outcomes for adulthood vary by race and gender [33]. For instance, Blacks have children at younger ages, while they marry at later ages, compared to Whites [32,34]. Therefore, this study provides understanding of whether and how the relationship between neighborhood disadvantage and health by race–gender status changes from 18 to 30 years old. 

### 1.1. Background and Theory

This research is guided by elements of the stress process model [35,36]. According to this theoretical framework, the conditions associated with neighborhood disadvantage, including poverty, high unemployment, physical disarray, and elevated crime rates, are conceptualized as stressors, which impact health outcomes by challenging the individual’s ability to adapt and cope [36,37]. These stressors create acute, immediate health challenges that, in turn, give way to chronic, longer-term health problems [35,36]. This process is referred to as stress proliferation, wherein primary stressors (e.g., few employment opportunities) spread to other areas of life and result in secondary stressors (e.g., lack of access to health care, untreated chronic conditions) [36,37]. In other words, neighborhood disadvantage has rippling effects that threaten health and maintain the disadvantage over time.

### 1.2. Neighborhoods, Race, Gender and Health 

A key feature of the stress process is the recognition that experiences that impact health differ by race and gender across the life course [12]. With regard to neighborhood stressors, research on health clearly indicates that the stressors that are linked to health and well-being vary by racial minority status (e.g., minorities experience higher rates of impoverishment and unemployment) and gender (e.g., women are more likely to shoulder the burden of rearing children alone in the context of economic insecurity) [38]. This research indicates the importance of how race and gender intersect to impact health [39,40]. For instance, both Black and White women experience more health inequality compared to men; however, Black women generally have worse health than White women [41,42]. With regard to poor neighborhood conditions, these racialized gender differences emerge early in the life course and may be due to the greater likelihood that Black women endure life in more economically fragile communities, live in areas with inadequate access adequate health care, and remain in disadvantaged neighborhoods because of the lack of residential mobility [43,44]. Therefore, carefully assessing how race and gender jointly impact health outcomes is one important goal of this research.

### 1.3. Neighborhood Disadvantage and Health in Young Adulthood

In young adulthood, the major benchmarks for achieving adult status are the completion of education, launching stable employment and income, establishing residency independent of the family of origin, marrying, and entering parenthood [45,46,47]. While these transitions are not characteristics of all young people and may happen in varying order, they offer many opportunities to set goals, exercise choice, and direct one’s life. All of these choices and transitions happen in the context of the neighborhoods in which the young adult lives [47]. Therefore, neighborhood disadvantage not only impacts the success of making the desired transitions, but also the psychological and physical well-being of young adults. To illustrate, if the individual resides in a high-crime community in which there are few employment or economic prospects, their health may suffer [48]. Unemployment is often accompanied by the inability to purchase health insurance and having limited means of transportation, which in turn challenges the ability to have access to quality health care [48,49]. Moreover, the likelihood to afford healthy foods, live in safe environments free of dilapidation, and enjoy spaces where exercise can occur is less in these disadvantaged neighborhoods [37,38,43]. Because racial minorities and women in young adulthood are more likely to live in neighborhoods with concentrated poverty, they are more likely to suffer the health and well-being consequences of community disadvantage than their White male counterparts are [39].

Gender socialization is another factor that may account for health differentials between young men and women. Gender ideals restrict both women and men, but in different ways. Women are constricted to ideals of femininity rooted in self-sacrifice and the nurturance of others, while men are encouraged to follow masculine notions in which they signal toughness, show a lack of emotional expression, and engage in questionable health behaviors (e.g., smoking and heavy drinking) [50,51,52]. Regardless of race, women are more likely to internalize stressors associated with neighborhood disadvantage [37]. Moreover, because socialization places them at the center of caring for family, especially minor children, the psychological and physical burdens connected to deprived neighborhood environments are more likely to wear on the health of women [37,38]. The weight of deprived neighborhood conditions may especially erode the health of Black women, who are not only more likely to live in poor communities, but also to be rearing their children as the sole breadwinner [53,54].

### 1.4. Summary and Hypotheses

The current study explores the relationship between neighborhood disadvantage and self-rated poor health for a nationally representative sample of Black and White young adults aged 18–30. Using representative longitudinal data, I investigate whether and how race–gender status matters for how poor neighborhood conditions impact health and whether age matters during young adulthood. Four hypotheses were developed for this study:

**Hypothesis** **1** **(H1).** *Neighborhood disadvantage is positively associated with poor health.*

**Hypothesis** **2a** **(H2a).** *The size of the association between neighborhood disadvantage and poor health is greater for Black women compared to Black men.*

**Hypothesis** **2b** **(H2b).** *The size of the association between neighborhood disadvantage and poor health is greater for White women compared to White men.*

**Hypothesis** **3** **(H3).** *Black women have the highest probability of poor health compared to all groups.*

**Hypothesis** **4** **(H4).** *Older age is associated with a larger association size between poor neighborhood conditions and poor health.*

## 2. Materials and Methods

### 2.1. Sample

I examine the relationship between neighborhood disadvantage and poor health using data from the National Longitudinal Study of Youth 1997 (NLSY97). The NLSY97 is a multiwave panel dataset of 8984 youths between the ages of 12 and 16 on 31 December 1996 [55]. The data include a wide range of measures ranging from health to family life to criminal justice involvement. Respondents were interviewed each year from 1997 to 2011, and biennially thereafter. For this study, I used data from 16 waves (1997–2013) of the NLSY97 and restricted the sample to those Black and White respondents who had at least two measures of the dependent variable, self-rated health. The analytic sample includes 2298 Blacks (1108 men and 1190 women) and 20,191 person-years, and 4522 Whites (2317 men and 2205 women) and 38,710 person-years.

All analyses below were weighted to maintain the national representation of the United States and correct for the oversampling of Black youth. To arrive at the final sample size, I conducted multiple imputations by chained equations for the less than 10% of respondents with missing data on our independent and control variables. Fifteen replicate datasets were imputed and analyzed, and results were pooled to arrive at the estimates presented below [56].

### 2.2. Measures

All variables in this study are time-varying and measured at each wave, with the exception of race and gender. Fair/poor self-rated health is the dependent variable. Respondents were asked to categorize their general health as excellent, very good, good, fair, or poor. I coded those who reported fair or poor health as 1 and compared them to all others. In the analyses and discussion below, this measure is referred to as “poor health”.

My key independent variable is an index of neighborhood disadvantage, which includes three dimensions: (1) the percentage of poor female-headed houses, (2) the unemployment rate, and (3) serious crime rate per 100,000 (e.g., murder, forcible rape, robbery) [2]. These three dimensions were captured by county-level census data available through a contract with the Bureau of Labor Statistics, which manages the NLSY97 collection. Further, each component (e.g., unemployment rate) was centered at zero with a standard deviation of 1 and then summed to create a measure of neighborhood disadvantage that ranges from 0 (lower disadvantage) to 3 (higher disadvantage). One key independent and moderating variable is age, measured in years, and centered at 18 for the analyses below. Other moderator variables included dummy variables to indicate race (Black (1 = yes) and White (1 = yes)) and gender (female (1 = yes)).

Several relevant control variables were selected, including marriage (1 = yes), parenthood (1 = yes), and employment (1 = yes), compared to those who do not hold these roles. Other controls include whether the respondent had been arrested (1 = yes) and/or incarcerated (1 = yes). I also held constant education (1 = college degree or more) and household income (1 = top one-fifth of the household income distribution). Lastly, I controlled for community location, including whether the respondent lived in the southern region of the United States (1 = yes) and/or in an urban area (1 = yes).

### 2.3. Analytic Strategy

All analyses for this research were conducted using SAS 9.4 (SAS Institute, Cary, NC, USA). The first step in the analyses was to produce descriptive statistics for all study variables by race (Table 1). Second, I estimated two sets (i.e., one set for Blacks and the other for Whites) of subsample regression models. Generalized estimating equations were employed with exchangeable correlation structure and the logistic link function to handle the correlated nature of the repeated measures in the data [29]. In the first set of models, I established the impact of neighborhood disadvantage, age, and gender status by race (Table 2, Models 1A and 2A). In the second model, neighborhood disadvantage, age, and gender interactions were used to test whether the effect of neighborhood disadvantage varies by age and race–gender status (Table 2, Models 1B and 2B).

## 3. Results

Table 1 shows the means and percentages for all study variables. Consistent with other nationally representative data, a greater percentage of Blacks (9.96%) reported poor health compared to Whites (6.29%). There were no differences in age across the two groups, but in the subsamples, there was a greater percentage of Black women (51.80%) compared to White women (48.77%). Further, Blacks (0.36) reported higher levels of neighborhood disadvantage compared to Whites (0.17). Blacks also reported lower levels of employment (59.96% vs. 72.79%) and marriage (9.59% vs. 19.04%), but higher levels of parenthood (41.57% vs. 21.95%), than their White counterparts did. Moreover, in comparison to Whites, fewer Blacks had completed college degrees or higher (6.76% vs. 14.06%) and fewer fell into the top one-fifth in family income (9.43% vs. 21.06%). Moreover, greater percentages of Blacks than those of Whites lived in urban areas (80.42% vs. 69.02%) and the South (58.80% vs. 29.67%). Lastly, higher percentages of Blacks had an arrest history (6.43% vs. 4.79%) and reported having been incarcerated (12.28% vs. 7.84%) compared to Whites. 

In Table 2, Models 1A and 2A show that the probability of poor health increases by odds of 4% and 8% with each year of age for Blacks and Whites, respectively. Further, Black women, compared to Black men, experience increased odds of 83% for poor health, while White women had increased odds of 46% compared to White men. Neighborhood disadvantage also increases the odds of poor health by 13% for Blacks and 4% for Whites. Additionally, employment, a college degree or more, and family income decreased the odds of poor health for both Blacks and Whites, while parenthood, a history of arrest, and incarceration increased the odds for both groups. For Blacks, southern residence decreased the odds of poor health. Among Whites, marriage decreased the odds of poor health, while urban residence and living in the South increased the odds of poor health.

In Table 2, Models 1B and 2B display the findings for how neighborhood disadvantage, age, and gender are jointly associated with the probability of poor health for Blacks and Whites. With the exception of the interactions added to these models, most covariates retain the same general effects as in Models 1A and 2A. For Blacks (Table 2, Model 1B), the interaction of age by female indicates that age results in greater decreased odds (*OR* = 0.74) for Black women compared to Black men. Further, the female by neighborhood disadvantage interaction indicates that poor neighborhood conditions are less nettlesome (*OR* = 0.74) for Black women than they are for Black men. However, these advantages for Black women are not outweighed by the increased odds associated with being female (*OR* = 4.56, *p* < 0.001), which indicates that Black women are still at greater risk for poor health in comparison to Black men.

**Figure 1:**
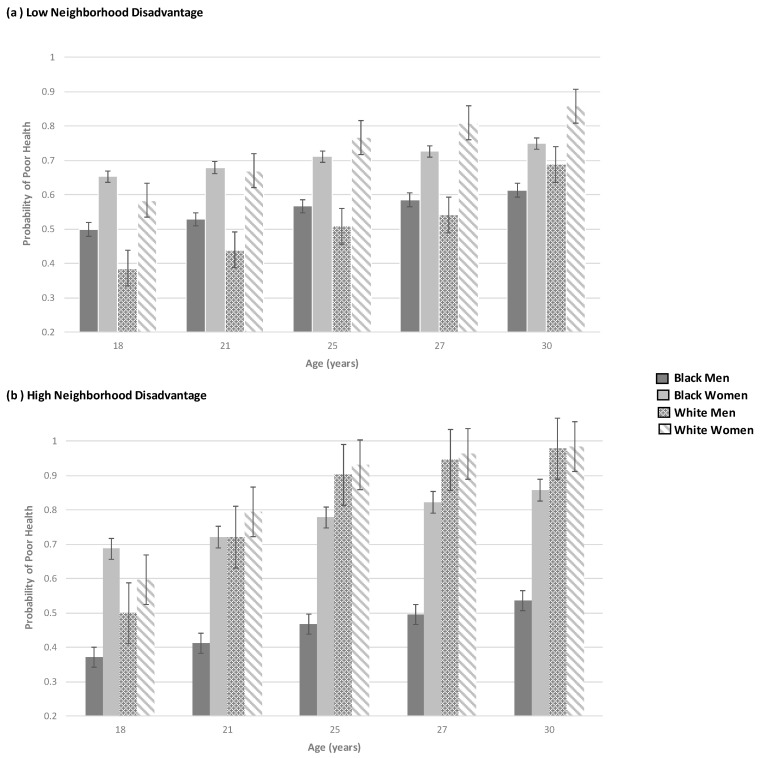
Relationship between poor health and race–gender status for those living in (**a**) low disadvantaged neighborhoods (bottom one-third) and (**b**) high disadvantaged neighborhoods (top one-third) at ages 18, 21, 25, 27, and 30.

For Whites (Table 2, Model 2B), White women experienced lower odds of poorer health as a result of the joint impact of age, gender, and neighborhood disadvantage (i.e., age × female × neighborhood disadvantage; *OR* = 0.88, *p* < 0.01) compared to White men. However, White women still had higher odds of poor health connected to aging (i.e., age × female; *OR* = 1.27, *p* < 0.01) and as a result of the increased odds being female (*OR* = 1.61, *p* < 0.05). The interaction findings are graphically displayed in Figure 1a,b.

In Figure 1a,b, neighborhood disadvantage is divided into low disadvantage (bottom one-third) and high disadvantage (top one-third) and includes age and race–gender status to allow both within and between race comparisons. Among those residents who live in communities with low neighborhood disadvantage (Figure 1a) at age 18, White men report the lowest probability of poor health followed by Black men who report the second lowest probability of poor health. At age 18, Black and White women have the highest probabilities of poor health, with Black women slightly higher as shown by the nearly overlapping error bars. At age 21, this pattern in low disadvantaged neighborhoods persists, with men having lower probabilities of poor health than those of women; however, by age 21, Black and White women do not differ significantly. As young adults mature to age 25, gender, not race, is the clearest driver of poor health. That is, Black and White men have equivalent probabilities of poor health, as do Black and White women. Nevertheless, by age 27, White women have the overall highest odds of poor health, followed by Black women. At age 30, White women still have poorer health than any other group. Black women do not differ significantly from White men, and Black men have the lowest overall probability of poor health. 

Figure 1b displays the findings for poor health by age and race–gender status for young adults in in high disadvantaged neighborhoods. At age 18, Black men have the lowest probability of poor health, while Black and White women do not differ significantly. However, White men have a lower probability of poor health than Black women do, but do not differ from White women. At ages, 21, 25, 27, and 30, the pattern that emerges is that Black men remain at the lowest odds of poor health; however, Black women, White women, and White men do not differ significantly. Comparing Figure 1a,b, two things are worth noting. First, as might be expected, the odds of poor health are overall lower in the neighborhoods with low disadvantage. Second, in both high and low disadvantage, age is positively associated with poor health. That is, regardless of race–gender status, the general pattern is that all groups report higher odds of poor health as they age.

## 4. Discussion

In this study, I investigated the association between neighborhood disadvantage and poor health. Important to the assessment of this main relationship was whether and how race, gender, and age matter in young adulthood. In H1, I hypothesized that neighborhood disadvantage would be positively associated with poor health and found support for this hypothesis. As neighborhood disadvantage increases, so does the probability of poor health. Interestingly, the results also reveal several important, unexpected nuances. For example, while H2, which stated that neighborhood conditions would generate higher odds of poor health for women compared to men, was supported for Blacks, it was not supported for Whites. In both low and high disadvantaged neighborhoods, Black women have greater odds of poor health. Black men had the lowest odds of poor health in most disadvantaged neighborhoods compared to all other groups. However, among Whites, women only had higher odds of poor health in low disadvantage neighborhoods. In communities with high levels of disadvantage, White men did not differ from White women in the probability of poor health. 

Further, I did not find support for H3, which stated that Black women would overall have the highest probability of poor health. In fact, in neighborhoods with low disadvantage, White women had the greatest odds of poor health, and in the most severely disadvantaged communities, there was no difference in the odds of poor health for Black and White women. H4 predicted that, as young adults age, the association between neighborhood disadvantage and poor health would grow. This hypothesis was supported for Whites, as shown by the significant interaction between age and neighborhood disadvantage. The indication is that, as Whites age across young adulthood, the impact of deprived neighborhood conditions worsens health. However, this “aging effect” is less severe for White women, as signified by the triple interaction (i.e., age × female × neighborhood disadvantage) in Table 2, Model 2B. 

This research was fruitful in at least two ways. First, I verified the importance of neighborhood disadvantage for individuals in young adulthood. While people in this stage of the life course are generally healthy, this research shows that health during this period is impacted by neighborhood circumstances. Moreover, the severity of poor health increase significantly for individuals aged from 18 to 30 years old, especially in the most highly disadvantaged neighborhoods. The probability of poor health is much greater for most groups, and the more disadvantages and disorders are endured by neighborhood residents, except for Black males—a point to which I return below. Health is dependent on many factors, including environmental (e.g., physical dilapidation), psychosocial adjustment (e.g., low fear of crime), and socioeconomic resources (e.g., employment, transportation) [2,12,37]. Such resources are in short supply in poor neighborhoods and result in deprivation that render many of the developmental tasks (e.g., individuating from the family origin, completing education, and finding steady work) of young adulthood difficult, if not impossible [39,45]. In addition to having less access to economic resources such as employment and health care in disadvantaged neighborhoods, the failure to achieve markers of adulthood may cause additional stress that further puts health at risk.

Second, the results in this study support and add to our theoretical knowledge of how the neighborhood context is connected to health. In particular, the stress process model purports that stressors such as neighborhood disadvantage may vary by race and gender status [35,36]. In other words, on the basis of specific experience to group membership (e.g., Black women), stressors may have differential effects on health across groups. In the current study, I found a health paradox with respect to Black Americans, neighborhood disadvantage, and poor health. A health paradox occurs when one group is more highly exposed to a known health stressor (e.g., neighborhood disadvantage), but has health outcomes that are equivalent or better than those of groups with lower exposure to the same stressor [29,57]. That is, as I hypothesized, one might have anticipated that Black men and women, who experience neighborhood disadvantage at much higher rates than those of their White counterparts, would have comparatively poorer health. However, in the most disadvantaged neighborhoods, Black women experienced health outcomes equivalent to White men and women. Moreover, Black men experienced the lowest probability of poor health in communities with the highest levels of disadvantage. 

Of course, this study is not the first to find a Black–White health paradox. To name a few areas, researchers interested in mental health, mortality, criminal justice involvement, and immigration have all found such health paradoxes [41,57,58,59]. The typical theoretical reasoning is that Blacks may have different coping strategies as a result of obstacles they face in their daily lives and owing to their history in U.S. (e.g., enslavement and exclusion), which has required a level of adaptation and adjustment not needed by other groups. In related work on health, Christie-Mizell and his colleagues [41] found a health paradox in which Black males do not suffer higher odds of poor health due to a history of arrests, even though they are more likely than White counterparts to be arrested or have other types of criminal justice involvement—stressors linked to poor health. They theorized beyond the initial encounter with the criminal justice system that Black men who have been arrested are far more likely than others to remain involved with the criminal justice system through restitution, probation, rehabilitation services (e.g., drug courts), and community service; see also [60,61]. As burdensome as this continued involvement may be for the individual, such supervision may promote health by curbing involvement in behaviors (e.g., alcohol and drug use) that harm health and may result further criminal justice involvement. They further posited that the fact that higher rates of arrest are unjustly foisted on Black men, these higher rates for may be viewed as a problem of the criminal justice system and thereby associated with less distress and stigma, which can harm health [41]. 

I similarly argue here regarding neighborhood disadvantage for the young adult Black men and women in this study. Because this group is more likely to live in disadvantaged neighborhoods, the stigma may also be less health-threatening than that for their White counterparts. Moreover, while Black and White individuals share the same developmental tasks in young adulthood, the patterns for accomplishing these tasks vary. For example, Blacks experience more unstable employment, later marriage, and earlier fertility than Whites do. Therefore, Black health may not be as compromised by neighborhood conditions that do not encourage the White middle-class normative developmental trajectories. Lastly, the greater likelihood that Blacks have intergenerationally lived in less desirable communities because of exclusion and segregation may shield health in ways not experienced by Whites. The intergenerational knowledge of how to cope and manage such environments may be more prevalent in disadvantaged communities that have had to find ways to survive. Nevertheless, the ability to cope in ways not experienced by Whites does not completely protect Blacks from neighborhood disadvantage. As shown in my analyses, poor neighborhood conditions are still significantly related to poorer health for Blacks, placing Black women among the race–gender groups with the highest levels of poor health in highly disadvantaged neighborhoods.

## 5. Conclusions

This research expands the knowledge of how neighborhood disadvantage is related to poor health for young adults. The findings reveal that even relatively healthy young people accrue health risks as they age through early adulthood. This risk is especially prominent in the most highly disadvantaged neighborhoods. Nevertheless, there are important differences by race and gender status that are worth noting. Blacks who live in the most highly disadvantaged neighborhoods seem to be somewhat shielded from the most deleterious effects of poor neighborhood conditions compared to their White counterparts. Despite greater proportions of Blacks residing in harsh neighborhood environments, Black men experience better health than all other groups, and the health of Black women is no worse than White men or women. These results provide and extend the stress process model’s acknowledgement that individual and group experience molds how stressors and stress proliferation vary by important characteristics such as race and gender. 

This study is limited in a few respects. First, although I measured neighborhood disadvantage over time, these data do not allow for us to know respondent perceptions of neighborhood conditions. Other research has found that both objective and subjective ratings of neighborhoods can reveal the nature and extent to which individuals have internalized the meanings of neighborhood conditions, thus clarifying the impact on health and well-being. Second, life course research indicates that the importance placed on social roles (e.g., employment, marriage, parenthood) is key to understanding health. The enactment of personally meaningful roles encourages health because individuals are more likely to adopt prosocial behavior, approach adversity with resilience, and develop effective coping strategies [2,35,37]. Therefore, data that include role expectations, aspirations, and meaning would be helpful in clarifying the impact of neighborhood disadvantage on health. Third, future research should further investigate the Black–White health paradox (i.e., higher residency in disadvantaged neighborhoods but better or equivalent health) and explore the mechanisms underlying this paradox and the extent to which it applies other, specific health measures (e.g., depression, anxiety, cardiovascular problems, insomnia). Lastly, I purposely restricted the current study to Black and White men and women, but other research should expand it to include other groups (e.g., Latinx, Asian, Native American) with an emphasis on how race and ethnicity intersect with gender to produce outcomes across groups.

## Figures and Tables

**Table 1:** Mean/proportions for all study variables by race ^a^. National Longitudinal Survey of Youth 1997 (NLSY97), 1997–2013 ^b^.

	Blacks		Whites	
Variables ^b^	Mean/%	Std.	Mean/%	Std.
Poor health	9.96%	––	6.29% *	––
Age (years)	22.55	3.06	22.47	3.04
Female (1 = yes)	51.80%	––	48.77% *	––
Neighborhood disadvantage	0.36	0.68	0.17 *	0.68
Employed (1 = yes)	59.96%	––	72.79% *	––
Married (1 = yes)	9.59%	––	19.04%	––
Parenthood (1 = yes)	41.67%	––	21.95% *	––
College degree or more (1 = yes)	6.76%	––	14.06% *	––
Family income (1 = top fifth)	9.43%	––	21.06%	––
Urban (1 = yes)	80.42%	––	69.02% *	––
South (1 = yes)	58.80%	––	29.67% *	––
Arrest history (1 = yes)	6.43%	––	4.79% *	––
Respondent’s incarceration (1 = yes)	12.28%	––	7.84% *	––
*N* (person-years)	20,191		38,710	

^a^ Reported sample sizes refer to number of person-years. Subsample *N*s comprised 2298 Blacks and 4522 Whites. ^b^ Asterisks denote significant differences between Blacks and Whites at * *p* < 0.001.

**Table 2:** Generalized estimating equations for poor health by race. National Longitudinal Survey of Youth 1997 (NLSY97), 1997–2013.

	**Blacks**				**Whites**			
	**Model 1A**		**Model 1B**		**Model 2A**		**Model 2B**	
**Variables ^a^**	**Odds Ratio**	**95% CI**	**Odds Ratio**	**95% CI**	**Odds Ratio**	**95% CI**	**Odds Ratio**	**95% CI**
Age (centered at 18)	1.04 ***	(1.02–1.05)	1.31	(0.87–1.98)	1.08 ***	(1.06–1.09)	0.85	(0.68–1.06)
Female (1 = yes)	1.83 ***	(1.70–1.97)	4.56 ***	(2.11–9.85)	1.46 ***	(1.34–1.57)	1.61 *	(1.52–1.72)
Neighborhood disadvantage (ND) ^a^	1.13 ***	(1.04–1.23)	1.40 **	(1.12–1.75)	1.04 *	(1.02–1.07)	1.05 *	(1.03–1.07)
Control Variables								
Employed (1 = yes)	0.87 *	(0.77–0.98)	0.87 *	(0.77–0.98)	0.85 **	(0.77–0.94)	0.86 **	(0.77–0.95)
Married (1 = yes)	0.95	(0.84–1.07)	0.95	(0.84–1.07)	0.74 ***	(0.68–0.80)	0.74 ***	(0.68–0.80)
Parenthood (1 = yes)	1.38 ***	(1.28–1.48)	1.38 ***	(1.28–1.48)	1.38 ***	(1.28–1.49)	1.38 ***	(1.29–1.49)
College degree or more (1 = yes)	0.37 ***	(0.30–0.45)	0.37 ***	(0.30–0.45)	0.28 ***	(0.24–0.32)	0.28 ***	(0.24–0.32)
Family income (1 = top fifth)	0.74 ***	(0.65–0.85)	0.74 ***	(0.65–0.85)	0.56 ***	(0.51–0.61)	0.56 ***	(0.52–0.62)
Urban residence (1 = yes)	0.92	(0.84–1.01)	0.92	(0.84–1.01)	1.09 **	(1.02–1.16)	1.09 **	(1.02–1.16)
Southern residence (1 = yes)	0.80 ***	(0.75–0.86)	0.80 ***	(0.75–0.86)	1.08 *	(1.01–1.15)	1.08 *	(1.01–1.15)
Arrest history (1 = yes)	1.29 ***	(1.13–1.48)	1.29 ***	(1.13–1.48)	1.53 ***	(1.36–1.71)	1.54 ***	(1.37–1.72)
Respondent’s incarceration (1 = yes)	1.24 ***	(1.11–1.38)	1.24 ***	(1.11–1.38)	1.80 ***	(1.64–1.97)	1.80 ***	(1.64–1.97)
Interactions								
Age × female	––	––	0.72 *	(0.53–0.98)	––	––	1.27 **	(1.16–1.40)
Age × ND	––	––	0.92	(0.81–1.06)	––	––	1.12 *	(1.02–1.27)
Female × ND	––	––	0.74 **	(0.60–0.91)	––	––	0.95	(0.78–1.17)
Age × female × ND	––	––	1.11	(0.94–1.32)	––	––	0.88 **	(0.82–0.93)
AIC	25,441.70		25,440.72		34,832.72		37,829.74	
−2 Log likelihood	−12,707.85		−12,704.36		−17,403.63		−17,397.73	

Note: *N* = 2298 Blacks with 20,191 observations, and 4522 Whites with 38,710 observations. ^a^ Neighborhood disadvantage ranges from 0 (lower disadvantage) to 3 (higher disadvantage). * *p* < 0.05; ** *p* < 0.01; *** *p* < 0.001.

## Data Availability

Data from this study are publicly available and can be retrieved through the U.S. Bureau of Labor Statistics at https://www.bls.gov/nls/nlsy97.htm (accessed 5 January 2022).
